# Pembrolizumab in a Patient with Treatment-Naïve Unresectable BRAF-Mutation Negative Anaplastic Thyroid Cancer

**DOI:** 10.1155/2021/5521649

**Published:** 2021-05-22

**Authors:** Fadi Nabhan, Elizabeth Kander, Rulong Shen, Amit Agrawal, Vineeth Sukrithan, Ye Zhou, Ashima Goyal, Katie Roll, Manisha Shah, Bhavana Konda

**Affiliations:** ^1^Division of Endocrinology, Diabetes, and Metabolism, The Ohio State University and Arthur G. James Cancer Center, Columbus, OH 43235, USA; ^2^Division of Medical Oncology, Department of Internal Medicine, The Ohio State University Comprehensive Cancer Center, Columbus, OH, USA; ^3^Department of Pathology, The Ohio State University Wexner Medical Center, The Ohio State University, Columbus, OH, USA; ^4^Department of Otolaryngology—Head and Neck Surgery, The Ohio State University James Cancer Hospital and Solove Research Institute, Columbus, OH, USA

## Abstract

Immune check point inhibitor (ICI) therapy can be a potentially effective salvage treatment for anaplastic thyroid cancer (ATC) with progression despite standard of care therapies. We report a case of unresectable treatment-naïve ATC showing a dramatic and durable response to first-line pembrolizumab therapy. A 69-year-old male presented with a large, right-sided neck mass associated with compressive symptoms. A neck ultrasound showed a large, right-sided, and highly suspicious thyroid nodule. A fine needle aspiration (FNA) biopsy revealed tumor cells consistent with ATC that were positive for PD-L1, with an expression score of >95% and negative for the BRAF V600E mutation. Imaging studies were negative for distant metastases. The disease was declared surgically inoperable, and the patient declined chemotherapy/radiation therapy (XRT), but agreed to ICI therapy with intravenous pembrolizumab 200 mg every three weeks. The patient has received 25 doses of pembrolizumab to date, with rapid resolution of symptoms and a significant reduction in tumor size. He remains alive without disease progression 18 months since initial diagnosis.

## 1. Introduction

ATC is a rare and aggressive cancer comprising 1.7% of all thyroid cancers [1] that disproportionately accounts for about one-half of thyroid cancer-related deaths [2]. The disease is uniformly lethal, with a median overall survival (OS) of 3-4 months [3]. However, recent reports indicate a trend towards improved survival that is attributed to the development of more effective therapies [4]. Many patients have advanced disease at the time of diagnosis, and 46% of patients had distant metastases at diagnosis in one study [5]. The frequent coexistence of ATA with well-differentiated thyroid cancer (DTC) in tumor specimens, in conjunction with the presence of several shared genomic alterations with DTC suggests that ATC can result from dedifferentiation of DTC. However, the genomic profile of ATC is more complex, and some appear to also arise without evidence of dedifferentiation from DTC [6]. Additionally, ATC has higher mutational burden and genomic disruption than DTC and poorly differentiated thyroid cancer (PDTC) reflecting the significantly higher aggressiveness [7]. Genomic disruption and the acquisition of additional mutations might make ATC an attractive target for immune check point inhibitor therapies de novo. Traditional treatment of ATC depends on the extent of disease at presentation. Stage IVA and resectable stage IVB disease are treated with surgery and external radiation with or without chemotherapy. Chemotherapy and radiation can be considered for unresectable stage IVB and stage IVC patients [2]. The recent FDA approval of dabrafenib (BRAF inhibitor) and trametinib (MEK inhibitor) in advanced BRAF V600E mutated ATC has provided an additional treatment option for this subset of patients [8]. However, progression of disease is frequent which calls for additional therapeutic options. While immunotherapy has been tried as a salvage treatment of patients with ATC [9, 10], there is limited data on its use as a first-line therapy [11]. Here, we report such a case and review-related literature.

## 2. Case

**Figure 1:**
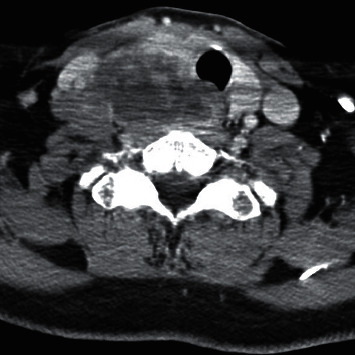
CT scan of the neck at presentation (axial image).

**Figure 2:**
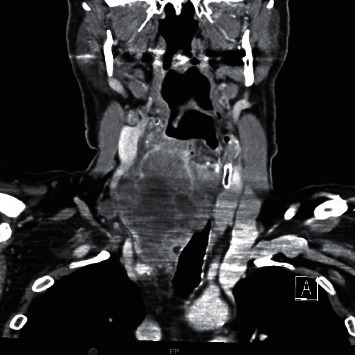
CT scan of the neck at presentation (coronal image).

**Figure 3:**
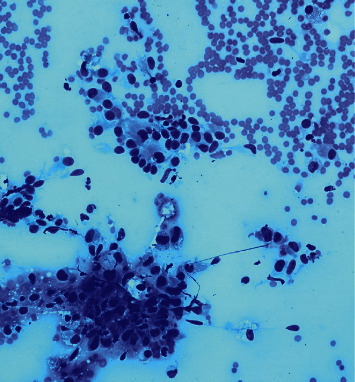
Cytology showing atypical discohesive cells consistent with ATC.

**Figure 4:**
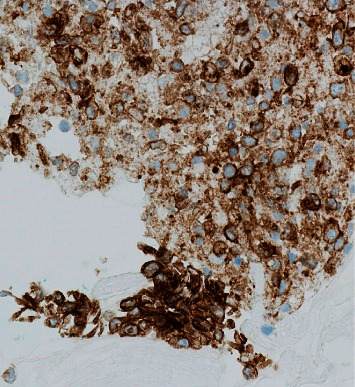
Immunocytochemistry staining showing strong positivity for PD-L1.

**Figure 5:**
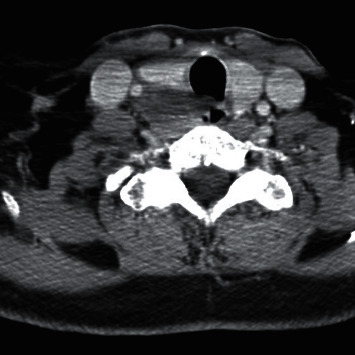
First CT scan of the neck after the initiation of pembrolizumab, 4 doses (axial image).

**Figure 6:**
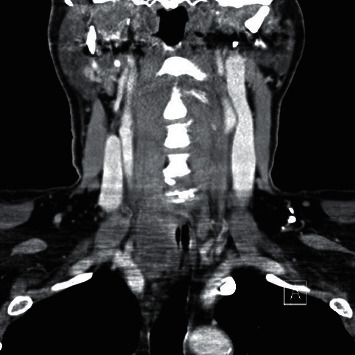
First CT scan of the neck after the initiation of pembrolizumab, 4 doses (coronal image).

**Figure 7:**
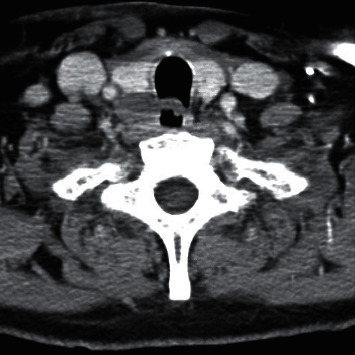
CT scan of the neck after 25 doses of pembrolizumab (axial image).

**Figure 8:**
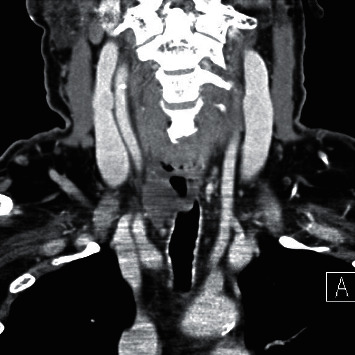
CT scan of the neck after 25 doses of pembrolizumab (coronal image).

A 69-year-old male presented with a 2-month history of dysphagia, dysphonia, pain in the right neck and right ear, and a ten-pound weight loss. Thyroid function tests were normal. A physical examination revealed a large, right-sided neck mass. Confirmed by a neck ultrasound, the mass was hypoechoic with macrocalcification and ill-defined borders measuring at least 5.5 cm. A fine needle aspiration (FNA) biopsy at an outside institution revealed malignancy best classified as primary thyroid cancer with high grade and spindle cell origin with suspicion of anaplastic thyroid cancer (ATC). The patient was then referred to our institution for further management. Laryngoscopy revealed right vocal cord paralysis. A CT scan of the neck showed a large, right thyroid lobe mass measuring 7.5 × 4.1 cm, displacing the larynx and trachea to the left and encasing the internal carotid artery by at least 180° (Figures 1 and 2 ). CT scans of the chest, abdomen, and pelvis and an MRI scan of the brain showed no definitive evidence of distant metastases. Serum thyroglobulin (Tg) level was at 41.5 ng/ml (Beckman Coulter assay with functional sensitivity of 0.1) with undetectable anti-Tg antibodies (Beckman Coulter assay with a functional sensitivity of 0.9). A repeat FNA biopsy of the right thyroid mass revealed discohesive atypical pleomorphic tumor cells consistent with ATC (Figure 3). Immunocytochemistry staining of the both FNA samples were focally positive for TTF 1, variably positive for thyroglobulin and PAX8, and negative for calcitonin, CEA, SOX-10, and p63. PD-L1 22C3 pharmDx—performed on Dako Autostainer Link 48—was positive for high PD-L1 expression with tumor proportion score (TPS) >95% (Figure 4). Molecular testing of the tumor was negative for BRAF (V600/601), KRAS (Exon 2/3/4), and NRAS mutations. Next generation sequencing of the tumor could not be done due to inadequate tissue. Liquid biopsy revealed no reportable genomic alterations. However, this was performed while the patient was on pembrolizumab treatment and not before its initiation. The tumor was deemed unresectable, and the patient declined chemotherapy and radiation therapy. Given the high PD-L1 expression, off-label intravenous pembrolizumab at 200 mg every three weeks was initiated with patient consent and absence of clinical trial accessible to the patient. One week after the first dose, the patient had significant improvement in dysphagia and resolution of pain in the right neck and ear. Restaging scans after four cycles of treatment (one cycle equaling three weeks) revealed a partial response per RECIST v 1.1 (48% decrease in the size of the tumor) (Figures 5 and 6 ). He continues to tolerate the treatment well without adverse events, except for the development of immune-mediated thyroiditis. After an initial thyrotoxic phase, the thyroiditis has progressed toward persistent hypothyroidism and has required thyroid hormone replacement therapy. The patient has received 25 doses of pembrolizumab with continued reduction in size of tumor with most recent scans performed showing approximately 66% reduction in tumor from pretherapy (Figures 7 and 8 ). The tumor has become resectable but with remaining significant potential high functional morbidity such as possible laryngopharyngectomy and tracheal and esophageal resection given its location. However, the patient continues to decline surgical therapy.

## 3. Discussion

The evolving understanding of the pathogenesis of ATC at the molecular level has provided insight into its management. ATC differs from well-differentiated thyroid cancer by having a higher mutation burden [7]. However, as stated before, many ATC arise from dedifferentiation of DTC. Genomically, ATC is considered to be composed of three types: one that resembles papillary thyroid cancer (PTC) and has a high prevalence of the *BRAF* V600E mutation; second that resembles follicular thyroid cancer (FTC) and has a high prevalence of the NRAS mutation; and third that has less identifiable genes and appears to resemble hurthle cell thyroid cancer (HCTC) [12]. In ATC patients with the BRAF V600E mutation, which is estimated to be present in 41% of patients [12], the U.S. Food and Drug Administration (FDA) approved the combination of BRAF plus MEK inhibitors (dabrafenib and trametinib, respectively). Subbiah and colleagues [8] reported 16 ATC patients with the BRAF V600E mutation that were treated with dabrafenib and trametinib with an overall response rate of 69% at a median follow-up of 47 weeks.

Immunotherapy has been tried as a treatment for ATC patients who have progressed on other therapies. Iyer and colleagues [9] evaluated 12 patients with ATC on combination therapy of pembrolizumab with tyrosine kinase inhibitors (TKI) where there was a progression of disease on TKI. With combination therapy and a median treatment of 5.6 months, there was a partial response in 42% (5/12) of patients and stable disease in 33% (4/12) of patients [9]. Cabanillas and colleagues [10] reported a case of a patient with ATC where pembrolizumab was used after progression of disease with chemotherapy and dabrafenib/trametinib. The treatment was for approximately ten months, with interruption due to surgery and external radiation, and the patient was alive 12 months after treatment [10]. An ongoing phase 2 study (ATLEP) of the combination of lenvatinib and pembrolizumab showed an interim response rate of 37.5% (6/16; all partial responses) with the rest experiencing stable disease [13]. A phase 2 study of spartalizumab, a PD-1 inhibitor, in ATC reported response rates of 19% (8/42) including three complete responses [14]. Another study of the combination of lenvatinib and pembrolizumab is currently ongoing (NCT04171622).

To the best of our knowledge, there is only one other reported case where ICI therapy was used as a primary treatment for a patient with inoperable and treatment-naïve ATC, with a high PD-L1 expression (60%) in tumor cells [11]. This patient had a near complete response but, however, developed grade 4 colitis after eight cycles of pembrolizumab, which required drug discontinuation, leading to subsequent progression and death 18 months after initial diagnosis [11].

PD-L1 expression has been shown to be as high as 75% in patients with ATC [15]. However, it is important to note that some of the PD-L1 expression may be related to the ensuing immune response to the tumor rather than as an independent process implicated in disease progression. Yet, the association of a worse prognosis with high PD-L1 expression [16] may reflect the latter. Additionally, PD-L1 staining in cytological specimens can be more challenging than in histological ones if the tumor is heterogeneous [17]. When the tumor is homogenous, there is an evidence that the detection and quantification of PD-L1 expression shows a high degree of concordance (>90%) between cytologic and histologic specimens in non-small cell lung cancer [18]. Whether a high PD-L1 expression score is predictive of treatment response in ATC remains unclear. In the present case and another case report [10], the PD-L1 expression was >95%, while the response was irrespective of the degree of PD-L1 expression in a case series by Iyer and colleagues [9].

## 4. Conclusion

ATC is an aggressive malignancy with a very low survival rate which frequently presents with distant metastases or unresectable disease [19]. This case highlights the possibility of using ICI therapy as a primary treatment modality in treatment-naïve unresectable ATC patients. However, it has yet to be determined whether ICI therapy may be used as a neoadjuvant treatment to allow for surgical intervention or if it may be used as a long-term maintenance therapy when other forms of treatment are refused or when surgery is not feasible. In addition, studies further exploring the use of ICIs as an alternative to chemoradiation are necessary. More studies of immune check point blockade in ATC [20] are in progress, and the results will provide valuable information on the optimal use of these medications in ATC.
